# Current epidemiology of chronic liver disease

**DOI:** 10.1093/gastro/goae069

**Published:** 2024-06-24

**Authors:** Richie Manikat, Aijaz Ahmed, Donghee Kim

**Affiliations:** Division of Gastroenterology and Hepatology, Department of Medicine, Stanford University School of Medicine, Stanford, CA, USA; Division of Gastroenterology and Hepatology, Department of Medicine, Stanford University School of Medicine, Stanford, CA, USA; Division of Gastroenterology and Hepatology, Department of Medicine, Stanford University School of Medicine, Stanford, CA, USA

**Keywords:** NAFLD, MASLD, hepatitis B, hepatitis C, alcohol-related liver disease

## Abstract

Chronic liver disease presents a significant global health burden, characterized by several etiologies, including metabolic dysfunction-associated steatotic liver disease (MASLD), alcohol-related liver disease (ALD), chronic hepatitis B virus infection, and chronic hepatitis C virus infection. This review explored current epidemiological trends and projections for each etiology, looking into their respective burdens and challenges. MASLD, formerly known as nonalcoholic fatty liver disease, is the most prevalent cause of chronic liver disease, and its global incidence and prevalence are steadily rising. ALD, fueled by increased alcohol consumption, is also on the rise, with concerning implications for future mortality rates. Chronic hepatitis B and C infections remain major public health concerns, particularly in specific regions of the world, necessitating concerted efforts for screening and treatment. The coronavirus disease 2019 (COVID-19) pandemic has impacted the epidemiology of chronic liver disease, exacerbating mortality rates and disrupting healthcare services. Mental health issues arising from the pandemic further complicate the treatment of chronic liver disease, making comprehensive healthcare strategies essential. Despite advancements in treatment, chronic liver disease continues to impose a substantial economic burden, emphasizing the importance of preventive measures and early intervention. In conclusion, ongoing surveillance and research efforts are crucial for understanding and addressing the evolving landscape of chronic liver disease. Comprehensive strategies that encompass prevention, screening, and treatment of its different etiologies are essential for mitigating its impact and improving patient outcomes.

## Introduction

Chronic liver disease is a sizable issue in global health, and the heterogeneity of its underlying causes poses significant challenges. The most common etiologies of chronic liver disease include metabolic dysfunction-associated steatotic liver disease (MASLD), alcohol-related liver disease (ALD), and chronic viral hepatitis, namely, chronic hepatitis B virus infection and chronic hepatitis C virus infection. A detailed understanding of the current epidemiological burdens and trends in these respective etiologies can be instructive to clinical practitioners and members of any discipline involved in helping individuals affected by chronic liver disease.

## MASLD

Nomenclature changes in hepatic steatosis were recently proposed and are currently being adopted by most stakeholders. The change from nonalcoholic fatty liver disease (NAFLD) to MASLD reflects the removal of the “nonalcoholic” label, enabling the inclusion of cardiometabolic criteria while dropping a potentially stigmatizing definition. A recent study has shown that there is minimal discrepancy between NAFLD and MASLD, and it is reasonable to consider that findings from NAFLD studies remain valid under the new definition of MASLD [1]. MASLD is the most common cause of chronic liver disease. This insidious condition (steatotic liver disease [SLD]) is diagnosed when fat accumulates in more than 5% of the liver or hepatic steatosis by imaging modality. MASLD was defined as the presence of SLD with one or more of the cardiometabolic criteria and no other well-known causes of SLD like significant alcohol consumption (>140 g/week in women and >210 g/week in men), other etiology, medications, genetic disorders, and starvation [2, 3]. Though emerging literature increasingly favors the use of MASLD over NAFLD, most of the available data regarding the epidemiology of MASLD still use the diagnostic criteria for NAFLD, which may be reflected throughout the rest of this review.

### Incidence and prevalence

Due to heterogeneity in data and coding diagnoses, estimating the incidence of MASLD has been challenging [4]. A meta-analysis of 12 studies reports a pooled incidence rate of 48.89 (95% confidence interval [CI]: 38.49–61.93) per 1,000 person-years in a sample size of 256,757 individuals [5]. The incidence of NAFLD increased by 58% from 37.41 per 1,000 person-years (95% CI: 24.36–57.06) in 1994–2006, 52.74 (95% CI: 36.15–76.34) in 2007–2008, to 59.11 per 1,000 (39.64–87.26) in 2010–2014 survey year. The contemporary prevalence of MASLD is staggering, and the manifestations range from simple steatosis to metabolic dysfunction-associated steatohepatitis (MASH), which may then further progress to advanced fibrosis and cirrhosis. According to one meta-analysis, MASLD has a global prevalence of 30.1% (95% CI: 27.9%–32.3%), and it has been rising over the past three decades [5]. The global prevalence of MASLD increased by 50% recently, from 25.3% (95% CI: 21.6%–29.3%) in 1990–2006 to 38.2% (95% CI: 33.7%–42.9%) in 2016–2019. This rise in MASLD correlates with the increased prevalence of obesity and diabetes, while the prevalence of chronic viral hepatitis continues to trend down [6].

The National Health and Nutrition Examination Survey (NHANES) collects a representative sample of the non-institutionalized civilian population in the United States. An analysis of the NHANES data has shown that the prevalence of MASLD is 34% in this population when hepatic ultrasound is used to detect mild to severe steatosis [7]. This prevalence predicts that 43.2 million Americans have underlying MASLD when extrapolated to the general population. A recent study using the NHANES 2017–2018 showed that the age-adjusted prevalence of SLD was 35.1% (95% CI: 32.0%–38.2%), as defined by transient elastography [8].

A global meta-analysis published in 2022 looked at 72 studies and 1,030,160 individuals and found a global MASLD prevalence of 32.4% [9], similar to the 30.1% reported by Younossi *et al.* [5], even though the sample size was smaller. The study by Riazi *et al.* [9] did not include individuals from the Middle East and Africa as the analyzing group did not find any studies that met their inclusion criteria, limiting a truly global representation of the conclusions.

Research has also investigated the prevalence of MASLD in specific populations. A recent study reported that nearly 70% of overweight individuals had MASLD, with the region of the Americas at 75% and the African region at 48%, demonstrating differences in various areas around the globe [10]. Even in persons without obesity, MASLD prevalence was found to be 40% [11]. Multiple other studies have addressed the heterogeneous and variable prevalence of this condition across geographic regions, race/ethnicity, and obesity status [12–15]. For example, in patients living with HIV, the prevalence of MASLD is 39%, according to a multicenter study conducted across several sites in the United States [16]. MASLD is also seen more commonly in South American populations than in other regions, with one study from Brazil reporting an MASLD prevalence of 35.2% [17]. Research is ongoing into the genes that predispose to the development of MASLD; however, the single nucleotide polymorphism rs738409 of the human patatin-like phospholipase domain-containing-3 (PNPLA3) gene appears to play a significant role in all populations and also accounts for the interethnic variability in disease susceptibility [17, 18]. Table 1 summarizes selected studies on the epidemiology of MASLD.

**Table 1.** Recent epidemiological studies of metabolic dysfunction-associated steatotic liver disease (MASLD)

Study	Country	Total population	Finding
Zou *et al.* (2019) [12]	10 countries	19 studies; 5,620 individuals	Overall pooled prevalence of MASLD in patients with IBD was 27.5%
Ye et al. (2020) [11]	24 countries	93 studies; 10,530,308 individuals	-Prevalence of nonobese MASLD among individuals with MASLD was 40.8%. -Prevalence of lean MASLD among individuals with MASLD was 19.2%.
de Vries *et al.* (2020) [13]	17 countries	20 studies; 3,901 patients with type 1 diabetes	Pooled MASLD prevalence in type 1 diabetes was 19.3%.
Riazi *et al.* (2022) [9]	17 countries	72 studies; 1,030,160 individuals	-Overall global MASLD prevalence was 32.4%. -Prevalence rose from 25.5% in or before 2005 to 37.8% in 2016 or later.
Le *et al.* (2022) [14]	25 countries	245 studies; 5,399,254 individuals	-Overall global MASLD prevalence was 29.8%. -Prevalence rose from 21.9% in 1991 to 37.3% in 2019, with annual increase of 0.7%.
Quek *et al.* (2023) [10]	35 countries	151 studies; 101,028 individuals	-MASLD prevalence in overweight population was 70.0%. -MASLD prevalence in obese population was 75.3%.
Hartmann *et al.* (2023) [15]	Worldwide	Data from Global Burden of Disease study on individuals aged 10 to 19 years	Global MASLD prevalence in adolescents rose from 3.73% in 1990 to 4.71% in 2019 (a relative increase of 26.27%).
Younossi *et al.* (2023) [5]	Worldwide	92 studies	-Global MASLD prevalence of 30.1%. -Prevalence increased by 50.4% from 25.3% in 1990–2006 to 38.2% in 2016–2019.

MASLD = metabolic dysfunction-associated steatotic liver disease, IBD = inflammatory bowel disease.

### Current trends and future projections

The prevalence of MASLD is expected to continue to rise, correlating with global increases in obesity and diabetes [19]. It remains to be seen whether the introduction of novel agents that treat MASLD will be able to reach a large enough population to curtail the projected rise substantially, though the prospect is tantalizing. In a placebo-controlled phase 3 clinical trial, an oral thyroid hormone receptor beta-specific agonist named resmetirom, which was approved by the Food and Drug Administration (FDA), was found to resolve MASH in nearly 30% of patients (29.9% in resmetirom 100 mg group vs 9.7% in placebo group, *P *<* *0.001) and improve fibrosis by at least one stage in about 25% of patients (25.9% in resmetirom 100 mg group vs 14.2% in placebo group, *P *<* *0.001) [20]. Newer medications are often subject to significant barriers in actually reaching patients, though, like cost, insurance-related barriers, and supply chain issues now exacerbated by the coronavirus disease 2019 (COVID-19) pandemic. Projection studies attempt to model the future and forecast that the total population with MASLD in the United States will be greater than 100 million by 2030, with a prevalence of 33.5% in those over age 15, assuming current trends continue unabated [21]. More concerningly, the incidence of decompensated cirrhosis may increase from 39,320 cases/year in 2015 to 105,430 cases/year in 2030, a jump of 168%, which raises a specter of significant morbidity and mortality to come, not to mention the additional stresses on the healthcare workforce and healthcare economy [21].

### Impact of COVID-19

The COVID-19 pandemic has had widespread effects on the epidemiology of MASLD. A recent US study using the National Vital Statistic System (NVSS) showed changes in mortality rates throughout the COVID-19 pandemic [22]. Quarterly age-standardized all-cause mortality due to MASLD was found to have steadily increased before the COVID-19 pandemic (quarterly percentage change [QPC]: 2.1%, 95% CI: 0.2%−2.9% for 2018 Q1–2019 Q4) and showed a faster rise in the early COVID-19 pandemic (QPC: 6.5%, 95% CI: 4.3%−9.2% for 2019 Q4–2020 Q4) [22]. Subsequently, mortality rates remained more stable, though long-term data in the post-COVID era are still lacking. Lifestyle choices may have become less prudent due to mental stress induced by the pandemic. Mental health has been shown to have deteriorated over the course of the pandemic, evidenced by greater rates of depression [23], which was closely related to MASLD [24] and MASLD-related mortality [25].

### Mortality

While usually an asymptomatic condition, MASLD has been associated with a higher risk of mortality. In one study analyzing NHANES data, individuals with MASLD had a 21% higher all-cause mortality than those without SLD with one or more criteria for cardiometabolic criteria (hazard ratio [HR]: 1.21, 95% CI: 1.11–1.31). However, increased cardiovascular mortality was not seen [26]. Interestingly, individuals with MetALD (MASLD with increased alcohol consumption) demonstrated a higher risk of all-cause (HR: 1.68, 95% CI: 1.10–2.57) and cancer-related mortality [27]. In a meta-analysis, liver-specific mortality in patients with MASLD was found to be 0.77 events per 1,000 person-years (95% CI: 0.33–1.77 events), while overall mortality was 15.44 per 1,000 person‐years (95% CI: 11.72–20.34 events) [28]. Patients with advanced fibrosis and/or MASH are at the highest risk for mortality, and this group of patients merits the closest monitoring, with targeted lifestyle modification strategies and other supportive measures [19, 27].

## ALD

### Incidence and prevalence

Mirroring the parallels seen between the rise in obesity and up-trending rates of MASLD, increased alcohol use may, in turn, portend rising rates of ALD. Early in the course of the COVID-19 pandemic, a cross-sectional study conducted in the United States found an increase of 29% in the number of drinks per day consumed and 21% higher rates of binge drinking when 993 individuals were surveyed in February 2020 and then again in April 2020 [29]. According to the National Institute on Alcohol Abuse and Alcoholism (NIAAA), 2019 to 2020 demonstrated the most considerable single-year increase in the per capita consumption of alcoholic beverages since 1968 [30]. An observational study analyzing data from the NHANES, the National Inpatient Sample (NIS), and the United Network for Organ Sharing (UNOS) registry found that while the overall prevalence of ALD appeared to remain stable from 8.8% in 2001–2002 to 8.1% in 2015–2016 (*P *=* *0.102), the proportion of patients with stage 3 fibrosis or greater and ALD increased significantly from 2.2% (95% CI: 0.4%–4.0%) to 6.6% (95% CI: 2.0%–9.9%; *P *=* *0.007) [31]. The UNOS data showed that from 2007 to 2017, the number of adult patients who were listed for liver transplantation with a diagnosis of ALD increased by 63%, while the number of patients who had both ALD and hepatocellular carcinoma increased by an astounding 178% [31]. Further information is needed to accurately quantify the degree of alcohol use in the general population, which can be confounded by inaccurate recall and lack of knowledge regarding standard drink sizes. Table 2 summarizes selected studies on the epidemiology of ALD.

**Table 2.** Studies on trends in mortality due to alcohol-related liver disease (ALD)

Study	Location	Study type	Population size	Finding
Kim *et al.* (2021) [32]	USA	Observational cohort, multicenter	867 patients with ALD	Presence of ALD was associated with higher overall mortality in patients with COVID-19 (HR 2.42, 95% CI: 1.29–4.55).
Marjot *et al.* (2021) [33]	Multinational	Cohort registry study	745 patients with ALD	ALD showed a positive association with death (OR 3.11; 95% CI: 2.12–4.55; *P *<* *0.001) and was found to be an independent risk factor for mortality after COVID-19 infection.
Yeo *et al.* (2022) [34]	USA	Cross-sectional national database (Optum Datamart)	25,721 patients with ALD	During COVID-19 (2020 Q2 and onward), 60- and 90-day mortality trended upward. By the end of 2020, about 1 in 4 patients with ALD from the emergency department and inpatient wards died by 90 days (2020 Q4, 90-day mortality = 24.75%) and 1 in 5 patients died by 60 days (60-day mortality = 21.19%).
Kim et al. (2022) [35]	USA	National database of death certificates (National Vital Statistic System)	213,888 deaths related to CLD	All-cause mortality due to ALD had been increasing at a quarterly rate of 1.1% before COVID-19, then sharply increased to a quarterly rate of 11.2% during COVID-19.

COVID-19 = coronavirus disease 2019, ALD = alcohol-related liver disease, HR = hazard ratio, CI = confidence interval, OR = odds ratio, CLD = chronic liver disease.

### Current trends and future projections

Recent trends in ALD are concerning and merit serious investigation. A modeling study was conducted based on data obtained from the Global Information System on Alcohol and Health maintained by the World Health Organization (WHO) and found that the per-capita consumption of alcohol increased globally from 5.9 L (95% CI: 5.8–6.1) in 1990 to 6.5 L (95% CI: 6.0–6.9) in 2017. Based on this trend, the proportion of persons consuming alcohol worldwide is expected to increase from 47% (95% CI: 44%–50%) up to 50% (95% CI: 46%–53%) in 2030, an annualized increase of 0.2% [36]. The authors partly speculate that this increase is related to increased economic wealth in low-income and middle-income countries and greater amounts of disposable income. Increased alcohol consumption globally is not without deleterious health effects and is likely to contribute to increased death rates directly. Another study published in 2020 used Markov modeling to predict mortality rates in 2040 and projects that deaths due to ALD will increase by 84%, from 8.23 (95% uncertainty interval [UI]: 7.92–9.29) per 100,000 person-years in 2019 to 15.2 (95% UI: 13.93–16.19) per 100,000 person-years in 2040 [37]. Rising alcohol use is hence of significant public health concern and should draw the attention of international and national authorities. Interestingly, the authors also predicted the impact of a hypothetical intervention similar to the combination of strategies implemented to combat tobacco use, which dropped rates of cigarette smoking in the United States by 75% over 40 years. If this hypothetical intervention decreased high-risk alcohol use by just 3.5% annually, then deaths caused by ALD would actually decrease by 7.6% in 2040 instead of increasing by 84% if no such intervention was implemented. Tobacco use was fought using several techniques, such as taxes on tobacco products, smoke-free policies that discouraged smoking in public places, public health campaigns, such as raising awareness of the drastic consequences like lung cancer, and support for tobacco cessation programs. Many of these tools should be adapted to combat alcohol use, as no one tool is likely to be successful on its own. As one of the few preventable causes of cirrhosis, ALD is a prime target for focused public health intervention with potential dramatic benefit to the general population. It remains to be seen if novel pharmacologic therapies, such as larsucosterol currently being studied for the treatment of alcohol-related hepatitis, will have a large enough impact on the disease course and reach enough people to turn the tide in this devastating disease [38, 39].

### Impact of COVID-19

Morbidity related to the increased use of alcohol is an area of academic interest. A recent study based on the US NIS dataset found that the rate of hospitalizations caused by ALD had been trending up steadily even before the pandemic. However, since COVID-19 became more widespread, this rate of hospitalizations may be accelerating [40]. Another study from Japan confirmed that hospitalization rates for ALD and pancreatitis were 1.2 times higher during the pandemic than in earlier periods [41]. On top of the worsened health-related problems inflicted on individuals, rising morbidity can also have a broader impact on time taken off work and may cause downstream economic effects on national and global productivity. Studies have also specifically looked at the mortality caused by ALD in the background of the COVID-19 pandemic. Following the trends in ALD-related hospitalizations, the mortality rates in ALD were found to have steadily increased by 1.1% every quarter from 2017 Q1 to 2020 Q1 prior to the pandemic in a study using data from the NVSS [35]. The QPC then jumped dramatically to 11.2% during the pandemic (95% CI: 6.7%–15.9% for 2020 Q1–2020 Q4).

### Mortality

As of 2019, approximately one-quarter of all deaths related to cirrhosis are estimated to be secondary to alcohol, according to the Global Burden of Disease Study [42]. ALD caused the greatest proportion of deaths due to cirrhosis in Europe (42%) and contributed the least in the Eastern Mediterranean area (8%). However, this data may underestimate the actual mortality rates, as countries with poor access to healthcare may not be able to collect enough data to capture the breadth of the issue entirely [43].

## Chronic hepatitis B

### Incidence and prevalence

Chronic hepatitis B currently remains the second most common etiology, leading to mortality in patients with cirrhosis [44]. As an area of intense public health concern, the WHO gathers data from national vital registration agencies and analyzes them regularly, publishing global hepatitis reports periodically. In 2022, there were 1.2 million new hepatitis B virus infections based on data from the WHO published in April 2024 [45]. A recent study estimated the global prevalence of hepatitis B to be 3.2% or a staggering 257.5 million people [46]. Nearly 70% of people with hepatitis B virus infection reside in sub-Saharan Africa or the Western Pacific region, highlighting the geographic disparities in prevalence and demonstrating the areas in greatest need of intervention [47]. In fact, half of the world’s hepatitis B virus infections are found in just three countries—China, India, and Indonesia. Table 3 summarizes selected studies on the epidemiology of chronic hepatitis B.

**Table 3.** Recent studies on the epidemiology of chronic hepatitis B

Study	Location	Study type	Population size	Finding
GBD 2019 Collaborators (2020) [42]	Worldwide	Estimation of disease-related death, incidence and prevalence based on data from WHO and national vital registration databases	World population	Mortality in 2019 was estimated to be 331,000 (95% CI: 279,000–392,000). Age-standardized death rate: 4 per 100,000 population (3.4–4.8).
Global Hepatitis Report (2017) [48]	Worldwide	Report on global prevalence	World population	Global prevalence of 3.5%, around 257 million people. 68% live in sub-Saharan Africa and the Western Pacific
Polaris Observatory Collaborators (2023) [43]	Worldwide	Report on global prevalence	World population	Global prevalence of 3.9%, around 292 million people. 80% live in Africa, the Western Pacific, and Southeast Asia.
Alberts et al. (2022) [49]	Worldwide	Meta-analysis of 520 articles	1,376,503 patients with cirrhosis	-Pooled prevalence of HBV among patients with cirrhosis was 59% in WHO Western Pacific region. -Pooled prevalence of HBV among patients with cirrhosis was 25% in WHO Southeast Asia region.
Im *et al.* (2022) [50]	Worldwide	Meta-analysis on occult HBV infection (HBsAg negative but HBV DNA positive)	140,521,993 individuals tested for HBV DNA (>99% blood donors)	-Prevalence of occult HBV infection was 0.98% (95% CI: 0.44–1.72) in high-endemicity countries and 12.0% (3.4–24.7) among high-risk individuals living in high-endemicity countries.
Sarin *et al.* (2020) [51]	Asia-Pacific region	Report on liver disease epidemiology in the Asia-Pacific region	Population of 3.6 billion in Asia-Pacific	-HBV caused 51.3% of all deaths due to cirrhosis in the Asia-Pacific region. -HBV caused 49.1% of all deaths due to liver cancer in the Asia-Pacific region.
Global Hepatitis Report (2024) [45]	Worldwide	Report on global prevalence	World population	Global prevalence of 254 million people. About 50% in people aged 30–54. About 12% in children aged under 18.
Polaris Observatory Collaborators (2024) [52]	25 countries	Report on estimated prevalence of hepatitis delta virus	95 million individuals positive for HBsAg	-Anti-HDV prevalence reported to be 2.0% in those positive for HBsAg. -Highest anti-HDV prevalence rate was in Mongolia (61%). -Highest absolute number of HDV RNA positive cases was in China (627,800).

CI = confidence interval, GBD = Global Burden of Disease, HBsAg = hepatitis B surface antigen, HBV = hepatitis B virus, HBV DNA = hepatitis B viral load, HDV = hepatitis delta virus, HDV RNA = hepatitis delta viral load.

Strategies for tackling this pervasive condition must be considered. Though there is no cure for hepatitis B, effective treatment with antivirals has been developed, with various professional societies providing detailed treatment guidelines targeting the patients most likely to benefit while minimizing side effects. The WHO conducted a meta-analysis and found that around 19%, or nearly 50 million people, of patients with hepatitis B virus infection, were eligible for treatment with antiviral agents, though the number of patients being treated is unfortunately far lower [53]. At the end of 2022, only about 2.6%, or around 7 million, patients with hepatitis B are actually being treated, pointing to significant issues with both accurate and widespread diagnosis and treatment [45].

### Current trends and future projections

In 2016, the World Health Assembly positioned eliminating hepatitis B virus infection by 2030 as a viable goal given current treatment and prophylaxis strategies despite the unavailability of a cure. A modeling study utilizing a Delphi process found that despite predicted downtrends in the incidence of hepatitis B virus infection, the number of deaths related to hepatitis B virus infection is actually expected to increase from 858,000 (95% UI: 646,000–1,301,000) in 2015 to 1,149,000 (860,000–1,718,000) in 2030 [43]. Another study based on the Global Burden of Disease found that only four countries (Namibia, Montenegro, Ireland, and Dominica) out of 194 had met the targeted 10% reduction in hepatitis B-related mortality, but 147 countries were able to achieve a 1% prevalence in children aged less than 5 years [54]. As most of the disease burden in hepatitis B derives from infections contracted before 5 years of age, the WHO has focused on this young population to curtail this condition in the long term [48]. Continued effort in the domain is likely to decrease the incidence of hepatitis B, though treatment strategies must continue to target those who have already contracted the infection, as well. Efforts in diagnosing people with hepatitis B are ongoing, and data have shown modest improvement over the years, with 10% of cases diagnosed in 2019 increasing to 13% diagnosed in 2022 [45].

### Impact of COVID-19

The COVID-19 pandemic has affected healthcare in nearly all specialties and phases of care, though its impact has been outsized in hepatitis B, which disproportionately affects lower-resource regions. COVID-19 was proven to negatively affect the progress in eliminating hepatitis B by hampering access to screening, testing, and treatment, according to a comprehensive survey administered to members of the European Association for the Study of the Liver (EASL) in 2021 [44, 55]. In the post-COVID-19 era, the public health infrastructure must work to both catch-ups and then continue to make progress if this infection is to be successfully contained and, eventually, eradicated.

### Economic burden

Though costs can be significant given the need for lifelong treatment in hepatitis B and annual monitoring of hepatitis B viral loads, increased testing and treatment do lead to overall cost savings realized by preventing the sequelae of chronic liver disease and decreased mortality rates, according to modeling studies conducted by the WHO [56].

### Mortality

Hepatitis B caused 1.1 million deaths in 2022, which increased from 820,000 deaths in 2019 [45]. This rise in mortality is thought to be due to the rising age of patients with hepatitis B compounded by the COVID-19 pandemic hampering access to treatment and follow-up [45]. Mirroring trends seen in prevalence, data from the WHO showed that 47% of the deaths occurred in the Western Pacific, 20% in Southeast Asia, and 25% in Africa, pointing to the areas in greatest need of intervention globally to staunch further mortality attributable to this infection.

In the United States, some signals point to decreasing mortality caused by viral hepatitis as a proportion overall of chronic liver disease-related mortality. One study showed that hepatocellular carcinoma-related mortality due to hepatitis B decreased by a QPC of 1.1% from 2017 to 2021 in adults aged 20 years and over [57]. Hepatitis B does, however, continue to be the leading cause of mortality related to liver cancer globally [58, 59].

### Hepatitis D virus

In some individuals with hepatitis B virus, the satellite RNA virus hepatitis D may also coexist. The global prevalence of anti-hepatitis D virus antibodies is around 2%, and the United States is estimated to have a prevalence of 3% [52]. Reflex testing, in which the laboratory automatically performs a test for anti-hepatitis D virus when a specimen is positive for the hepatitis B surface antigen, is felt to be an effective strategy to increase the early detection of patients with hepatitis D virus [52].

## Chronic hepatitis C

The hepatitis C virus causes chronic inflammation in the liver, leading to cirrhosis and hepatocellular carcinoma. Due to the threat it poses to public health, the WHO has envisioned a global approach to reduce the incidence and associated mortality of hepatitis C by increasing awareness, ramping up testing, and enabling broader access to direct antiviral therapy [60]. As the only major etiology of chronic liver disease that has a cure, hepatitis C is an enticing target for all those involved in promoting health.

### Incidence and prevalence

Half of the world’s hepatitis C infections are found in just six countries—China, India, Indonesia, Pakistan, the Russian Federation, and the United States of America [45]. About 71 million people are living with chronic hepatitis C, and the incidence in 2022 was 1 million new cases [45]. Table 4 summarizes selected studies on the epidemiology of chronic hepatitis C. Worldwide, the most common cause of spread is contaminated needles, which transmit the blood-borne infection. In the United States, for example, 23% of new infections and a third of deaths due to hepatitis C are seen in people who inject drugs (PWID) [63]. Because of the inherent stigma, this population is vulnerable and frequently experiences barriers to accessing testing and treatment for viral infections like hepatitis C [64]. Without dedicated effort in reaching this group, PWID will continue to experience disproportionate morbidity and mortality due to hepatitis C while also inadvertently contributing to the spread of the virus.

**Table 4.** Recent studies on the epidemiology of chronic hepatitis C

Study	Location	Study type	Population size	Finding
GBD 2019 Collaborators (2020) [42]	Worldwide	Estimation of disease-related death, incidence and prevalence based on data from WHO and national vital registration databases	World population	Mortality in 2019 estimated to be 395,000 (95% CI: 336,000–459,000). Age-standardized death rate: 4.8 per 100,000 pop (4.1–5.6).
Global Hepatitis Report (2017) [48]	Worldwide	Report on global prevalence	World population	Global prevalence of 71 million people, around 1%. Highest prevalence in WHO Eastern Mediterranean and European regions.
Jin *et al.* (2021) [61]	Worldwide	Meta-analysis of 194 publications	213,856 men who have sex with men	-Pooled prevalence of HCV in MSM was 3.4% (95% CI: 2.8–4.0). -Highest pooled HCV prevalence was 5.8%. (2.5–10.4) in Africa, then 5.0% (0.0–16.6) in Southeast Asia.
Sarin *et al.* (2020) [51]	Asia-Pacific region	Report on liver disease epidemiology in the Asia-Pacific region	Population of 3.6 billion in Asia-Pacific	-HCV caused 15.7% of all deaths due to cirrhosis in the Asia-Pacific region. -HCV caused 10.8% of all deaths due to liver cancer in the Asia-Pacific region.
Alberts *et al.* (2022) [49]	Worldwide	Meta-analysis of 520 articles	1,376,503 patients with cirrhosis	-Pooled prevalence of HCV among patients with cirrhosis was 13% in WHO Western Pacific region. -Pooled prevalence of HCV among patients with cirrhosis was 29% in WHO Southeast Asia region.
Polaris Observatory Study (2022) [62]	Worldwide	Estimate on global prevalence	World population	Global prevalence of 0.7%, around 57 million people. Prevalence highest in Eastern Europe and Central Asia.
Global Hepatitis Report (2024) [45]	Worldwide	Report on global prevalence	World population	Global prevalence of 50 million people. Incidence in 2022 was 1.0 million. 36% of patients with hepatitis C have been diagnosed and 20% have received curative treatment.

CI = confidence interval, GBD = Global Burden of Disease, HCV = hepatitis C virus, MSM = men who have sex with men, WHO = World Health Organization.

### Current trends and future projections

The introduction of direct-acting antiviral (DAA) therapy allowed for the cure of hepatitis C, a remarkable development in the history of medicine. Though the progress made in curing patients who have been diagnosed with hepatitis C should be acknowledged and continued, broad screening should not be relegated to a lower priority as that would run the risk of creating a large undiagnosed population. The asymptomatic nature of chronic hepatitis C makes underdiagnosis easy. Often, patients may experience financial challenges, substance use disorders, and mental health issues that are more urgent in their minds than the diagnosis of a hidden infection with adverse health consequences several years downstream [65]. Only with thorough and repeated education can the dangers of hepatitis C be clearly related to populations at risk.

The ongoing opioid epidemic, interruptions caused by COVID-19, and the continued use of DAAs make accurate predictions about the future of hepatitis C complicated and problematic. Efforts have been made, though, and one study using Markov modeling estimates that the incidence of chronic hepatitis C will be around 1.4 million every year until 2030 and that liver-related deaths will increase from 257,000 in 2020 to 290,000 in 2030 [62]. Continued and widespread use of DAAs remains essential in curtailing this virus and Egypt has made remarkable strides in this regard. More than a third of all patients initiated on DAA therapy from 2015 to 2019 were located in Egypt (3.5 million out of 9.5 million), and this country is expected to see lower rates of hepatocellular carcinoma and liver-related deaths in the near future as a result [62]. This degree of national resolve and resource allocation is vital in combating hepatitis C in other hard-hit countries while also proving that the approach of mass population-level screening, public awareness campaigns, and affordable, accessible treatment is effective.

### Impact of COVID-19

The COVID-19 pandemic has delayed international efforts to fight the spread of hepatitis C. In Italy, for example, a program to implement birth screening for viral hepatitis has been delayed, and all screening programs were paused in March 2020 in Egypt [66]. A modeling study estimated that a one-year delay in 2020 would cause an excess 44,800 (95% UI: 43,800–49,300) cases of hepatocellular carcinoma and an excess 72,300 (95% UI: 70,600–79,400) liver-related deaths from 2020 to 2030 when compared to a scenario where there was no such delay [66]. Public health authorities will need to consider these delays when planning viral elimination programs while considering innovative strategies. The pandemic may provide opportunities to improve care if effective action is taken. In Italy, one group instituted point-of-care HCV antibody tests bundled with rapid COVID-19 testing in persons above 50 years of age in northern Italy, finding 3% of the tested population positive for hepatitis C, with less than half of those positive being aware of their HCV infection [67]. Consolidating diagnostics in this manner can effectively mitigate care gaps caused by the COVID-19 pandemic.

### Hospitalizations and economic burden

Though overall decreasing the number of hospitalizations over time, hepatitis C still contributes to nearly a third of hospitalizations (31.6% [95% CI: 31.3%–31.9%]) related to chronic liver disease in the United States, according to a study analyzing the NIS from 2012 to 2016 [68]. The high costs associated with the use of DAA therapy have been thought to contribute to delays in treating patients diagnosed with hepatitis C. A modeling study was done, which estimated the cost of testing and treatment globally at $41.5 billion; however, the total economic benefit would be to the tune of $22.7 billion when increased productivity is taken into account [69]. Over a 10- to 20-year timeframe, DAA therapy has been found to be cost-saving, both from a healthcare perspective and from a patient perspective, due to less time needing to be taken off work [70].

### Mortality

Chronic hepatitis C has been shown to cause mortality by various mechanisms, most commonly through the development of end-stage liver disease and by increasing the risk of liver cancer. By some estimates, hepatitis C increases the risk of developing hepatocellular carcinoma by up to 20 times and remains a significant cause of hepatocellular carcinoma in the United States and globally [63, 71]. Mortality due to hepatitis C is also worsened by the concomitant rise in coexisting obesity and MASH. Encouragingly, however, the DAA era has been associated with an overall decline in cirrhosis-related mortality due to hepatitis C, according to a study analyzing information from the United States Census and the NVSS [72]. These trends should redouble efforts to expand access to lifesaving DAA medications globally, especially in the hardest-hit low- and middle-income countries.

## Conclusions

Recent epidemiologic trends should guide the actions of international and national health authorities. While viral hepatitis as a threat becomes less fearsome in some areas globally, in other parts of the world, it remains a significant danger, requiring the conscious dedication of resources and personnel. MASLD and ALD are even more difficult to tackle than hepatitis B and C in some regards, given their multifactorial causation and lack of a single effective pharmacotherapeutic agent; however, a lack of action may lead to further incidence akin to an epidemic. Close monitoring with continued observational studies of the different etiologies contributing to chronic liver disease remains a crucial facet to assessing the needs of different populations and the effectiveness of interventions already in play, which are constantly evolving.

## Authors’ Contributions

R.M., A.A., and D.K. were involved in the study concept and design, acquisition of data, interpretation of data, and drafting of the manuscript drafting and revising the manuscript. All authors read and approved the final manuscript.

## Funding

None.

## References

[1] Song SJ , Lai JCT, Wong GLH et al Can we use old NAFLD data under the new MASLD definition? J Hepatol 2024;80:e54–6–e56. 37541393 10.1016/j.jhep.2023.07.021

[2] Rinella ME , Neuschwander-Tetri BA, Siddiqui MS et al AASLD practice guidance on the clinical assessment and management of nonalcoholic fatty liver disease. Hepatology 2023;77:1797–835. 36727674 10.1097/HEP.0000000000000323 PMC10735173

[3] Rinella ME , Lazarus JV, Ratziu V et al A multisociety Delphi consensus statement on new fatty liver disease nomenclature. J Hepatol 2023;79:1542–56. 37364790 10.1016/j.jhep.2023.06.003

[4] Murag S , Ahmed A, Kim D. Recent epidemiology of nonalcoholic fatty liver disease. Gut Liver 2021;15:206–16. 32921636 10.5009/gnl20127 PMC7960978

[5] Younossi ZM , Golabi P, Paik JM et al The global epidemiology of nonalcoholic fatty liver disease (NAFLD) and nonalcoholic steatohepatitis (NASH): a systematic review. Hepatology 2023;77:1335–47. 36626630 10.1097/HEP.0000000000000004 PMC10026948

[6] Konyn P , Ahmed A, Kim D. Causes and risk profiles of mortality among individuals with nonalcoholic fatty liver disease. Clin Mol Hepatol 2023;29:S43–57. 36417893 10.3350/cmh.2022.0351 PMC10029952

[7] Kim D , Kim WR, Kim HJ et al Association between noninvasive fibrosis markers and mortality among adults with nonalcoholic fatty liver disease in the United States. Hepatology 2013;57:1357–65. 23175136 10.1002/hep.26156 PMC3622816

[8] Kim D , Cholankeril G, Loomba R et al Prevalence of fatty liver disease and fibrosis detected by transient elastography in adults in the United States, 2017-2018. Clin Gastroenterol Hepatol 2021;19:1499–501.e2. 32801011 10.1016/j.cgh.2020.08.017

[9] Riazi K , Azhari H, Charette JH et al The prevalence and incidence of NAFLD worldwide: a systematic review and meta-analysis. Lancet Gastroenterol Hepatol 2022;7:851–61. 35798021 10.1016/S2468-1253(22)00165-0

[10] Quek J , Chan KE, Wong ZY et al Global prevalence of non-alcoholic fatty liver disease and non-alcoholic steatohepatitis in the overweight and obese population: a systematic review and meta-analysis. Lancet Gastroenterol Hepatol 2023;8:20–30. 36400097 10.1016/S2468-1253(22)00317-X

[11] Ye Q , Zou B, Yeo YH et al Global prevalence, incidence, and outcomes of non-obese or lean non-alcoholic fatty liver disease: a systematic review and meta-analysis. Lancet Gastroenterol Hepatol 2020;5:739–52. 32413340 10.1016/S2468-1253(20)30077-7

[12] Zou ZY , Shen B, Fan JG. Systematic review with meta-analysis: epidemiology of nonalcoholic fatty liver disease in patients with inflammatory bowel disease. Inflamm Bowel Dis 2019;25:1764–72. 30918952 10.1093/ibd/izz043

[13] de Vries M , Westerink J, Kaasjager KHAH et al Prevalence of nonalcoholic fatty liver disease (NAFLD) in patients with type 1 diabetes mellitus: a systematic review and meta-analysis. J Clin Endocrinol Metab 2020;105:3842–53. 32827432 10.1210/clinem/dgaa575 PMC7526735

[14] Le MH , Yeo YH, Li X et al 2019 Global NAFLD prevalence: a systematic review and meta-analysis. Clin Gastroenterol Hepatol 2022;20:2809–17.e28. 34890795 10.1016/j.cgh.2021.12.002

[15] Hartmann P , Zhang X, Loomba R et al Global and national prevalence of nonalcoholic fatty liver disease in adolescents: an analysis of the global burden of disease study 2019. Hepatology 2023;78:1168–81. 37021791 10.1097/HEP.0000000000000383 PMC10521800

[16] Gawrieh S , Vilar-Gomez E, Woreta TA et al Prevalence of steatotic liver disease, MASLD, MetALD and significant fibrosis in people with HIV in the United States. Aliment Pharmacol Ther 2024;59:666–79. 38158589 10.1111/apt.17849 PMC10922859

[17] Yip TC-F , Vilar-Gomez E, Petta S et al Geographical similarity and differences in the burden and genetic predisposition of NAFLD. Hepatology 2023;77:1404–27. 36062393 10.1002/hep.32774

[18] Shen JH , Li YL, Li D et al The rs738409 (I148M) variant of the PNPLA3 gene and cirrhosis: a meta-analysis. J Lipid Res 2015;56:167–75. 25378656 10.1194/jlr.M048777 PMC4274064

[19] Cotter TG , Rinella M. Nonalcoholic fatty liver disease 2020: the state of the disease. Gastroenterology 2020;158:1851–64. 32061595 10.1053/j.gastro.2020.01.052

[20] Harrison SA , Bedossa P, Guy CD et al; MAESTRO-NASH Investigators. A phase 3, randomized, controlled trial of resmetirom in NASH with liver fibrosis. N Engl J Med 2024;390:497–509. 38324483 10.1056/NEJMoa2309000

[21] Estes C , Razavi H, Loomba R et al Modeling the epidemic of nonalcoholic fatty liver disease demonstrates an exponential increase in burden of disease. Hepatology 2018;67:123–33. 28802062 10.1002/hep.29466 PMC5767767

[22] Kim D , Manikat R, Wijarnpreecha K et al Chronic liver disease-related mortality during the COVID-19 pandemic. Eur J Intern Med 2023;118:129–32. 37777423 10.1016/j.ejim.2023.09.024

[23] Renaud-Charest O , Lui LMW, Eskander S et al Onset and frequency of depression in post-COVID-19 syndrome: a systematic review. J Psychiatr Res 2021;144:129–37. 34619491 10.1016/j.jpsychires.2021.09.054 PMC8482840

[24] Kim D , Dennis BB, Cholankeril G et al Association between depression and metabolic dysfunction-associated fatty liver disease/significant fibrosis. J Affect Disord 2023;329:184–91. 36841305 10.1016/j.jad.2023.02.101

[25] Kim D , Manikat R, Shaikh A et al Depression in nonalcoholic fatty liver disease and all-cause/cause-specific mortality. Eur J Clin Invest 2024;54:e14087. 37638383 10.1111/eci.14087

[26] Kim D , Wijarnpreecha K, Cholankeril G et al Metabolic dysfunction-associated steatotic liver disease and all-cause/cause-specific mortality among adults in the United States. J Hepatol 2024;80:e79–81. 37717601 10.1016/j.jhep.2023.09.014

[27] Kim D , Wijarnpreecha K, Cholankeril G et al Steatotic liver disease-associated all-cause/cause-specific mortality in the United States. Aliment Pharmacol Ther 2024;60:33–42. 38649335 10.1111/apt.18011

[28] Younossi ZM , Koenig AB, Abdelatif D et al Global epidemiology of nonalcoholic fatty liver disease—meta‐analytic assessment of prevalence, incidence, and outcomes. Hepatology 2016;64:73–84. 26707365 10.1002/hep.28431

[29] Barbosa C , Cowell AJ, Dowd WN. Alcohol consumption in response to the COVID-19 pandemic in the United States. J Addict Med 2021;15:341–4. 33105169 10.1097/ADM.0000000000000767 PMC8327759

[30] Slater M , Alpert H. *National Institute on Alcohol Abuse and Alcoholism | Surveillance Report #119*. 2022. https://pubs.niaaa.nih.gov/publications/surveillance119/CONS20.htm (10 April 2024, date last accessed).

[31] Dang K , Hirode G, Singal AK et al Alcoholic liver disease epidemiology in the United States: a retrospective analysis of 3 US databases. Off J Am Coll Gastroenterol ACG 2020;115:96. 10.14309/ajg.0000000000000380 31517639

[32] Kim D , Adeniji N, Latt N et al Predictors of outcomes of COVID-19 in patients with chronic liver disease: US multi-center study. Clin Gastroenterol Hepatol 2021;19:1469–79.e19. 32950749 10.1016/j.cgh.2020.09.027 PMC7497795

[33] Marjot T , Moon AM, Cook JA et al Outcomes following SARS-CoV-2 infection in patients with chronic liver disease: an international registry study. J Hepatol 2021;74:567–77. 33035628 10.1016/j.jhep.2020.09.024 PMC7536538

[34] Yeo YH , Zou B, Cheung R et al Increased mortality of patients with alcohol-related liver diseases during the COVID-19 pandemic in the United States. J Intern Med 2022;292:837–9. 35869603 10.1111/joim.13545 PMC9350353

[35] Kim D , Alshuwaykh O, Dennis BB et al Trends in etiology-based mortality from chronic liver disease before and during COVID-19 pandemic in the United States. Clin Gastroenterol Hepatol 2022;20:2307–16.e3. 35811045 10.1016/j.cgh.2022.05.045 PMC9262655

[36] Manthey J , Shield KD, Rylett M et al Global alcohol exposure between 1990 and 2017 and forecasts until 2030: a modelling study. Lancet 2019;393:2493–502. 31076174 10.1016/S0140-6736(18)32744-2

[37] Julien J , Ayer T, Bethea ED et al Projected prevalence and mortality associated with alcohol-related liver disease in the USA, 2019–40: a modelling study. Lancet Public Health 2020;5:e316–23–e323. 32504584 10.1016/S2468-2667(20)30062-1

[38] Pimienta M , Tien C, Terrault NA. Prospective clinical trials and novel therapies in the medical management of severe alcohol-associated hepatitis. Clin Liver Dis 2022;20:202–8. 10.1002/cld.1265 PMC9745256 36523864

[39] Hassanein T , McClain CJ, Vatsalya V et al Safety, pharmacokinetics, and efficacy signals of larsucosterol (DUR-928) in alcohol-associated hepatitis. Am J Gastroenterol 2024;119:107–15. 37011138 10.14309/ajg.0000000000002275 PMC10758349

[40] Kim D , Perumpail BJ, Wijarnpreecha K et al Trends in aetiology-based hospitalisation for cirrhosis before and during the COVID-19 pandemic in the United States. Aliment Pharmacol Ther 2023;58:218–28. 37189243 10.1111/apt.17547

[41] Itoshima H , Shin J h, Takada D et al The impact of the COVID-19 epidemic on hospital admissions for alcohol-related liver disease and pancreatitis in Japan. Sci Rep 2021;11:14054. 34253741 10.1038/s41598-021-92612-2 PMC8275590

[42] Vos T , Lim SS, Abbafati C et al; GBD 2019 Diseases and Injuries Collaborators. Global burden of 369 diseases and injuries in 204 countries and territories, 1990–2019: a systematic analysis for the Global Burden of Disease Study 2019. Lancet 2020;396:1204–22. 33069326 10.1016/S0140-6736(20)30925-9 PMC7567026

[43] Huang DQ , Mathurin P, Cortez-Pinto H et al Global epidemiology of alcohol-associated cirrhosis and HCC: trends, projections and risk factors. Nat Rev Gastroenterol Hepatol 2023;20:37–49. 36258033 10.1038/s41575-022-00688-6 PMC9579565

[44] Huang DQ , Terrault NA, Tacke F et al Global epidemiology of cirrhosis–aetiology, trends and predictions. Nat Rev Gastroenterol Hepatol 2023;20:388–98. 36977794 10.1038/s41575-023-00759-2 PMC10043867

[45] World Health Organization. *Global Hepatitis Report 2024: Action for Access in Low- and Middle-Income Countries*. https://www.who.int/publications-detail-redirect/9789240091672 (10 April 2024, date last accessed).

[46] Razavi-Shearer D , Gamkrelidze I, Pan C et al; Polaris Observatory Collaborators. Global prevalence, cascade of care, and prophylaxis coverage of hepatitis B in 2022: a modelling study. Lancet Gastroenterol Hepatol 2023;8:879–907. 37517414 10.1016/S2468-1253(23)00197-8

[47] Lim JK , Nguyen MH, Kim WR et al Prevalence of chronic hepatitis B virus infection in the United States. Off J Am Coll Gastroenterol ACG 2020;115:1429. 10.14309/ajg.0000000000000651 32483003

[48] World Health Organization. Global Hepatitis Report 2017. Geneva: World Health Organization, 2017, 83. https://iris.who.int/handle/10665/255016 (10 April 2024, date last accessed).

[49] Alberts CJ , Clifford GM, Georges D et al Worldwide prevalence of hepatitis B virus and hepatitis C virus among patients with cirrhosis at country, region, and global levels: a systematic review. Lancet Gastroenterol Hepatol 2022;7:724–35. 35576953 10.1016/S2468-1253(22)00050-4 PMC9259503

[50] Im YR , Jagdish R, Leith D et al Prevalence of occult hepatitis B virus infection in adults: a systematic review and meta-analysis. Lancet Gastroenterol Hepatol 2022;7:932–42. 35961359 10.1016/S2468-1253(22)00201-1 PMC9630161

[51] Sarin SK , Kumar M, Eslam M et al Liver diseases in the Asia-Pacific region: a Lancet Gastroenterology & Hepatology Commission. Lancet Gastroenterol Hepatol 2020;5:167–228. 31852635 10.1016/S2468-1253(19)30342-5 PMC7164809

[52] Polaris Observatory Collaborators. Adjusted estimate of the prevalence of hepatitis delta virus in 25 countries and territories. J Hepatol 2024;80:232–42. 38030035 10.1016/j.jhep.2023.10.043

[53] Tan M , Bhadoria AS, Cui F et al Estimating the proportion of people with chronic hepatitis B virus infection eligible for hepatitis B antiviral treatment worldwide: a systematic review and meta-analysis. Lancet Gastroenterol Hepatol 2021;6:106–19. 33197397 10.1016/S2468-1253(20)30307-1 PMC7801814

[54] Sheena BS , Hiebert L, Han H et al; GBD 2019 Hepatitis B Collaborators. Global, regional, and national burden of hepatitis B, 1990–2019: a systematic analysis for the Global Burden of Disease Study 2019. Lancet Gastroenterol Hepatol 2022;7:796–829. 35738290 10.1016/S2468-1253(22)00124-8 PMC9349325

[55] Kondili LA , Buti M, Riveiro-Barciela M et al Impact of the COVID-19 pandemic on hepatitis B and C elimination: an EASL survey. JHEP Rep 2022;4:100531. 35967191 10.1016/j.jhepr.2022.100531 PMC9364666

[56] Tordrup D , Hutin Y, Stenberg K et al Cost-effectiveness of testing and treatment for hepatitis B virus and hepatitis C virus infections: an analysis by scenarios, regions, and income. Value Health 2020;23:1552–60. 33248510 10.1016/j.jval.2020.06.015 PMC7806510

[57] Kim D , Manikat R, Cholankeril G et al Trends in mortality of liver cancer before and during the COVID-19 pandemic. Liver Int 2023;43:1865–70. 37387517 10.1111/liv.15668

[58] Choi S , Kim BK, Yon DK et al Global burden of primary liver cancer and its association with underlying aetiologies, sociodemographic status, and sex differences from 1990-2019: A DALY-based analysis of the Global Burden of Disease 2019 study. Clin Mol Hepatol 2023;29:433–52. 36597018 10.3350/cmh.2022.0316 PMC10121317

[59] Konyn P , Ahmed A, Kim D. The current trends in the health burden of primary liver cancer across the globe. Clin Mol Hepatol 2023;29:358–62. 36916167 10.3350/cmh.2023.0092 PMC10121285

[60] World Health Organization. *Developing Global Health Sector Strategies on HIV, Viral Hepatitis and STIs 2022–2030*. https://www.who.int/teams/global-hiv-hepatitis-and-stis-programmes/strategies/global-health-sector-strategies/developing-ghss-2022-2030 (10 April 2024, date last accessed).

[61] Jin F , Dore GJ, Matthews G et al Prevalence and incidence of hepatitis C virus infection in men who have sex with men: a systematic review and meta-analysis. Lancet Gastroenterol Hepatol 2021;6:39–56. 33217341 10.1016/S2468-1253(20)30303-4

[62] Blach S , Terrault NA, Tacke F et al; Polaris Observatory HCV Collaborators. Global change in hepatitis C virus prevalence and cascade of care between 2015 and 2020: a modelling study. Lancet Gastroenterol Hepatol 2022;7:396–415. 35180382 10.1016/S2468-1253(21)00472-6

[63] Dennis BB , Naji L, Jajarmi Y et al New hope for hepatitis C virus: Summary of global epidemiologic changes and novel innovations over 20 years. World J Gastroenterol 2021;27:4818–30. 34447228 10.3748/wjg.v27.i29.4818 PMC8371499

[64] Lazarus JV , Roel E, Elsharkawy AM. Hepatitis C virus epidemiology and the impact of interferon-free hepatitis C virus therapy. Cold Spring Harb Perspect Med 2020;10:a036913. 31570385 10.1101/cshperspect.a036913 PMC7050577

[65] Pedrana A , Munari S, Stoové M et al The phases of hepatitis C elimination: achieving WHO elimination targets. Lancet Gastroenterol Hepatol 2021;6:6–8. 33308435 10.1016/S2468-1253(20)30366-6

[66] Blach S , Kondili LA, Aghemo A et al Impact of COVID-19 on global HCV elimination efforts. J Hepatol 2021;74:31–6. 32777322 10.1016/j.jhep.2020.07.042 PMC7411379

[67] Wade AJ , Doyle JS. Interventions to eliminate hepatitis C: which ones suit your community? Lancet Gastroenterol Hepatol 2022;7:383–4. 35303491 10.1016/S2468-1253(22)00010-3

[68] Hirode G , Saab S, Wong RJ. Trends in the burden of chronic liver disease among hospitalized US adults. JAMA Netw Open 2020;3:e201997. 32239220 10.1001/jamanetworkopen.2020.1997 PMC7118516

[69] Scott N , Kuschel C, Pedrana A et al A model of the economic benefits of global hepatitis C elimination: an investment case. Lancet Gastroenterol Hepatol 2020;5:940–7. 32730785 10.1016/S2468-1253(20)30008-X

[70] Mattingly TJ , Slejko JF, Onukwugha E et al Value in hepatitis C virus treatment: a patient-centered cost-effectiveness analysis. Pharmacoeconomics 2020;38:233–42. 31788751 10.1007/s40273-019-00864-8 PMC7081653

[71] McGlynn KA , Petrick JL, El-Serag HB. Epidemiology of hepatocellular carcinoma. Hepatology 2021;73(Suppl 1):4–13. 10.1002/hep.31288 PMC7577946 32319693

[72] Kim D , Li AA, Gadiparthi C et al Changing trends in etiology-based annual mortality from chronic liver disease, from 2007 through 2016. Gastroenterology 2018;155:1154–63.e3. 30009816 10.1053/j.gastro.2018.07.008 PMC6467699

